# Efficacy and Safety of Stent-Assisted Coiling Versus Coiling Alone for Ruptured Multilobed Aneurysms: A Multicenter Study

**DOI:** 10.1161/SVIN.126.002556

**Published:** 2026-09-03

**Authors:** ZhenKun Xiao, ShanShan Mi, Wang Bing, YongHong Duan, AiHua Liu

**Affiliations:** Hengyang Key Laboratory of Hemorrhagic Cerebrovascular Disease, Department of Neurosurgery, the Second Affiliated Hospital, Hengyang Medical School, University of South China, Hunan (Z.X., S.M., W.B., Y.D., A.L.).; Beijing Neurosurgical Institute, Capital Medical University, China (A.L.).; Department of Interventional Neuroradiology, Beijing Tiantan Hospital, China (A.L.).

**Keywords:** hemodynamics, intracranial aneurysms, ischemic stroke, stents, subarachnoid hemorrhage

## Abstract

**BACKGROUND:**

Ruptured multilobed intracranial aneurysms present significant procedural challenges and high recurrence risks in endovascular treatment due to complex geometry and turbulent hemodynamics. Currently, the safety and long-term efficacy of stent-assisted coiling (SAC) versus coiling alone in treating this high-risk morphological subgroup remain clinically controversial; high-quality, large-scale evidence is required.

**METHODS:**

Based on a multicenter, prospective registry study (the SANE trial [Subarachnoid Aneurysm Endovascular Cohort Study in China]), 627 consecutive patients with ruptured multilobed intracranial aneurysm treated with SAC or coiling alone were included. Baseline demographics, perioperative safety outcomes (including intraprocedural thrombosis, ischemic stroke, cerebral vasospasm, and rerupture), clinical functional outcomes (modified Rankin Scale score and mortality), and angiographic morphological outcomes (Raymond-Roy Occlusion Classification) were systematically collected. Overlap weighting (average treatment effect in the overlap population) and doubly robust logistic regression models were applied to evaluate the independent clinical effects of different modalities after strict adjustment for confounders, including anatomic morphology and perioperative parameters.

**RESULTS:**

The cohort comprised 173 patients treated with coiling alone and 454 treated with SAC. The primary average treatment effect in the overlap population analysis included 610 patients and yielded a pooled Kish effective sample size of 438.74. Before weighting, perioperative ischemic stroke and intraprocedural thrombosis were more frequent with SAC. After weighting, all 16 prespecified covariates had absolute standardized mean differences <0.10. At follow-up, Raymond-Roy Occlusion Classification class I occlusion was observed in 75.8% of the SAC group and 53.9% of the coiling-alone group (*P*<0.001). Doubly robust models detected no statistically significant associations between SAC and the evaluated safety outcomes, poor functional outcome at discharge or follow-up, or all-cause mortality (all *P*≥0.071). SAC was associated with lower odds of follow-up Raymond-Roy Occlusion Classification class II versus I (odds ratio, 0.401 [95% CI, 0.203–0.795]; *P*=0.012) and class III versus I (odds ratio, 0.583 [95% CI, 0.307–0.806]; *P*=0.014).

**CONCLUSIONS:**

In this observational ruptured multilobed intracranial aneurysm cohort, SAC was associated with more favorable follow-up angiographic occlusion than coiling alone, without detected differences in perioperative safety or clinical outcomes. These findings do not establish functional superiority and remain subject to residual confounding.

**REGISTRATION:**

URL: https://www.chictr.org.cn/; Unique identifier: ChiCTR2000032657.

## Graphical abstract

**Figure:**
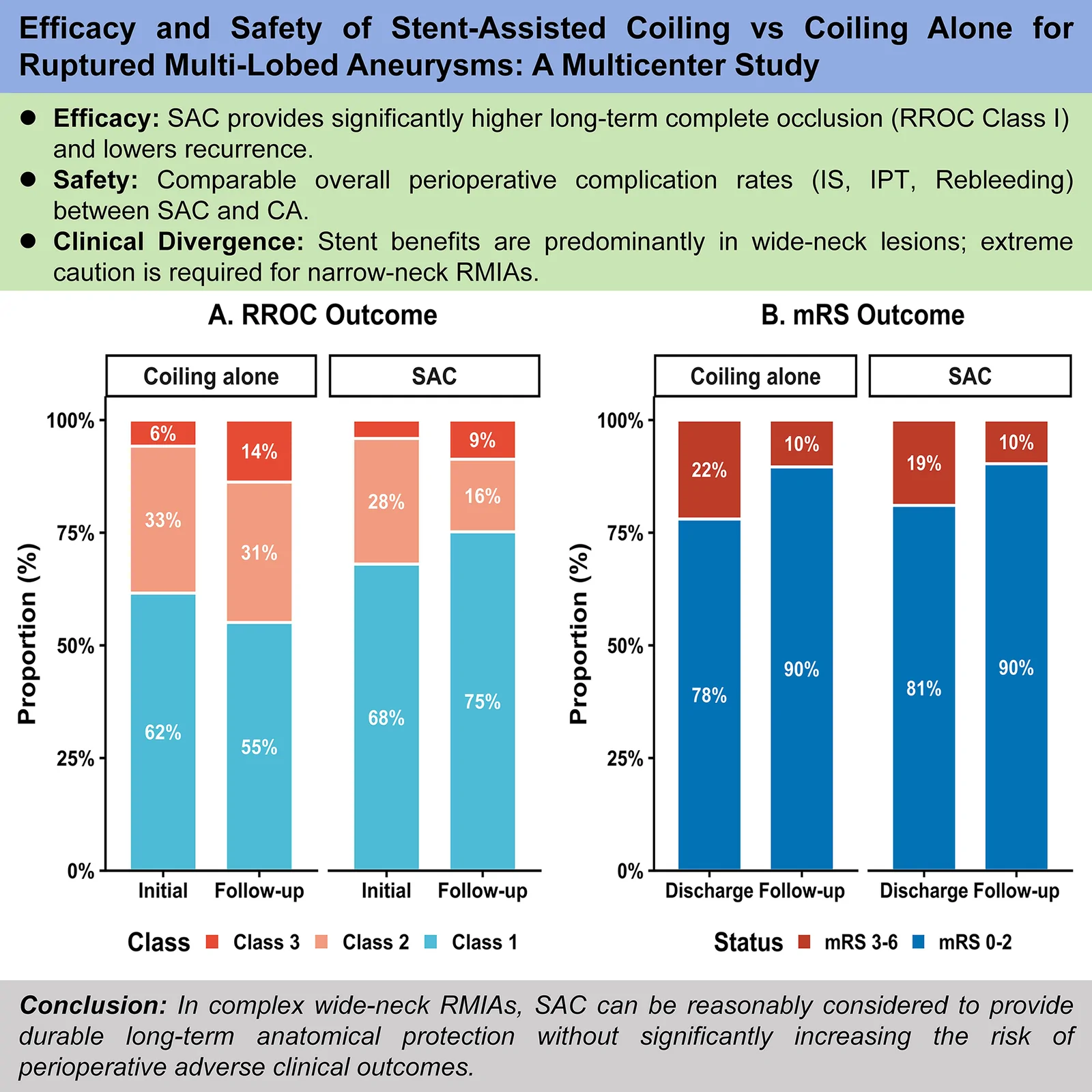

**CLINICAL PERSPECTIVE**

**What Is New?**

- Leveraging a large multicenter registry and advanced causal inference modeling, this study establishes that stent-assisted coiling for ruptured multilobed intracranial aneurysms significantly mitigates long-term morphological recurrence compared with coiling alone, without inflating overall perioperative complication rates. Notably, morphological stratification demonstrates that these durable angiographic benefits and superior long-term occlusion rates achieved by stent-assisted coiling are predominantly concentrated within the wide-neck lesion subgroup.

**What Are the Clinical Implications?**

- For highly irregular ruptured multilobed intracranial aneurysms presenting with wide-neck features, stent-assisted coiling should be reasonably prioritized as a definitive endovascular strategy to offer robust, long-term anatomic protection under rigorous perioperative antithrombotic management.

Aneurysmal subarachnoid hemorrhage (aSAH) is a severe cerebrovascular emergency.^1,2^ Research demonstrates that the 30-day mortality rate for aSAH typically ranges between 30% and 40%, and nearly half of survivors experience long-term neurological deficits or an inability to regain independent living status.^2^ Early intervention to occlude the ruptured aneurysm and prevent catastrophic rebleeding is a core principle for reducing early mortality and improving clinical outcomes.^3^

Based on evidence from large-scale randomized controlled trials, endovascular coiling has become a well-established treatment for ruptured intracranial aneurysms. Among patients considered suitable for either approach, coiling is associated with a lower risk of death or dependency than neurosurgical clipping.^4^ Among routine endovascular techniques, coiling alone is frequently utilized during the acute phase of aSAH. This approach does not require the intensive perioperative antiplatelet therapy generally needed for permanent intracranial stent implantation.^4,5^ However, a principal limitation of coiling alone is the risk of aneurysm recanalization and subsequent retreatment. Long-term follow-up studies have reported recurrence or reintervention rates approaching 30% in selected cohorts.^6^ To address unfavorable aneurysm anatomy that may limit stable coil retention, the application of stent-assisted coiling (SAC) has expanded.^7^ By providing intraluminal support across the aneurysm neck, SAC reduces the risk of coil protrusion into the parent artery and may promote progressive aneurysm occlusion. Comparative cohorts have suggested that SAC may improve the durability of occlusion, with recurrence rates of ≈15% reported in selected series.^7^ Despite these potential angiographic benefits, the use of SAC in acute aSAH requires antiplatelet therapy. Large cohort studies have reported clinically important thromboembolic and hemorrhagic complications after SAC for ruptured aneurysms.^8^ These findings underscore the need for careful patient selection and rigorous antiplatelet therapy management. Consequently, the choice between coiling alone and SAC remains a subject of clinical controversy.

Local aneurysm geometry is an important factor associated with rupture presentation and may also influence treatment selection. Lindgren et al^9^ demonstrated in a large-scale morphological analysis that irregular or multilobular aneurysm morphology was strongly associated with rupture across aneurysm sizes. This association was independent of aneurysm location and patient background.^9^ Owing to their complex sac geometry, the interventional treatment of ruptured multilobed intracranial aneurysms (RMIA) may pose significant technical challenges, including difficulty in achieving uniform coil distribution, stable neck coverage, and complete occlusion. The optimal endovascular strategy for RMIA remains highly debated. In aneurysms with unfavorable neck anatomy, SAC provides a scaffold for coil retention and may improve the durability of aneurysm occlusion.^8^ However, a meta-analysis by Zhang et al^10^ indicated that the use of SAC in ruptured aneurysms remains associated with clinically relevant hemorrhagic and thromboembolic concerns related to antiplatelet therapy. Nevertheless, recent evidence suggests that carefully selected tirofiban-based regimens may represent a feasible perioperative antiplatelet strategy, although hemorrhagic and thromboembolic risks remain clinically important.^11,12^ Despite the technical difficulty and recurrence risk associated with RMIA, the 2023 American Heart Association/American Stroke Association guideline for the management of aSAH does not provide treatment recommendations specifically for this morphological subgroup.^13^ In the existing literature, high-quality clinical cohort studies directly comparing angiographic and clinical outcomes between SAC and coiling alone in RMIA remain scarce.

Based on these clinical considerations, the present study used data from a multicenter, prospective registry (the SANE trial [Subarachnoid Aneurysm Endovascular Cohort Study in China]) to compare angiographic, perioperative safety, and clinical outcomes between SAC and coiling alone in RMIA. Specifically, the study evaluated the associations of treatment strategy with long-term incomplete occlusion or aneurysm recurrence, perioperative complications, and long-term functional outcomes.

## Methods

### Ethics Statement

The SANE trial is a national, investigator-initiated, prospective, consecutive, multicenter registry study. The study protocol was approved by the ethics committee of Beijing Tiantan Hospital, Capital Medical University (approval no. KY 2021-038-02) and was conducted in strict accordance with the Declaration of Helsinki and relevant ethical regulations for medical research in China. Written informed consent was obtained from all participants or their legally authorized representatives following a comprehensive explanation of the study objectives, treatment protocols, and potential risks and benefits.

### Data Sources and Setting

The SANE trial was a multicenter, prospective registry study involving neurovascular centers across 33 hospitals in 20 provinces in China. The primary objective of the SANE trial was to compare the efficacy and safety of SAC versus coiling alone in patients with aSAH. The SANE trial protocol and written informed consent were approved by the ethics committees and relevant regulatory authorities of all participating hospitals. Treatment modality was selected by the treating neurointerventional team after integrated assessment of the 3-dimensional aneurysm configuration, neck morphology, parent artery and branch anatomy, technical feasibility, intraoperative coil stability, hemorrhagic risk, and the anticipated need for adjunctive support. SAC was considered when coiling alone was technically unstable or unlikely to achieve satisfactory occlusion; however, the final treatment decision remained at the discretion of the treating operator. All procedures were performed by neurointerventionalists holding the rank of Chief Physician, each of whom had independently completed >500 neurointerventional procedures and performed >100 procedures annually.

For this secondary analysis, further ethical approval was not required as the data had been deidentified. This secondary analysis is reported in accordance with the STROBE guidelines (Strengthening the Reporting of Observational Studies in Epidemiology); full ethical review documents are provided in the Supplemental Material. This study adheres to the American Heart Association Journals’ implementation of the Transparency and Openness Promotion guidelines. The data that support the findings of this study are available from the corresponding author on reasonable request.

### Study Population

In this secondary analysis, only patients radiographically identified with RMIA who underwent either SAC or coiling alone were included. Schematic representations of RMIA and the 2 treatment modalities are provided in Figure 1. To ensure cohort homogeneity and minimize potential confounding effects, patients were excluded based on the following predefined criteria: (1) presence of multiple intracranial aneurysms where the ruptured culprit lesion could not be definitively identified; (2) a prior history of stroke; and (3) use of other endovascular modalities, including flow diverters, Woven EndoBridge devices, or overlapping stent techniques. Missing angiographic follow-up did not constitute an exclusion criterion; patients remained in the full cohort and contributed to analyses for which the relevant outcome data were available. A detailed flow chart of inclusion and exclusion is presented in Figure 2.

**Figure 1.**
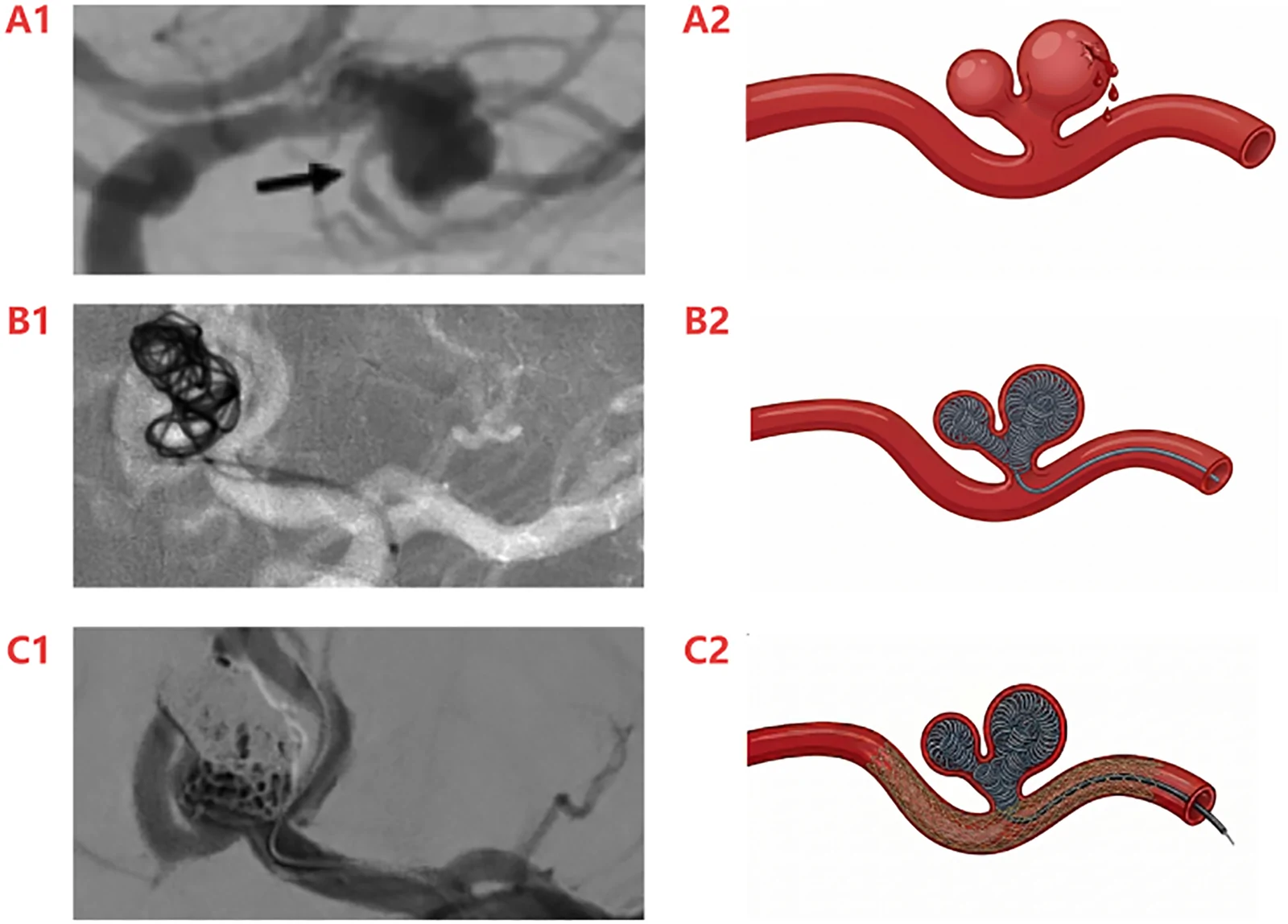
**Stent-assisted coiling (SAC) for ruptured multilobed intracranial aneurysm (RMIA).** Representative angiographic and corresponding schematic illustrations of SAC for an RMIA. **A1** and **A2**, Preprocedural 3-dimensional (3D) digital subtraction angiography (DSA) and 3D schematic model displaying the irregular multilobed geometry and wide-neck configuration of the RMIA. **B1** and **B2**, Intraprocedural 2-dimensional–DSA and illustration demonstrating initial microcatheterization and coil embolization before stent deployment. **C1** and **C2**, Final postprocedural angiogram and schematic confirming complete occlusion (Raymond-Roy Occlusion Classification class I); the successfully deployed stent secures the coil mass within the irregular sac, preventing coil herniation while ensuring the patency of the parent artery.

**Figure 2.**
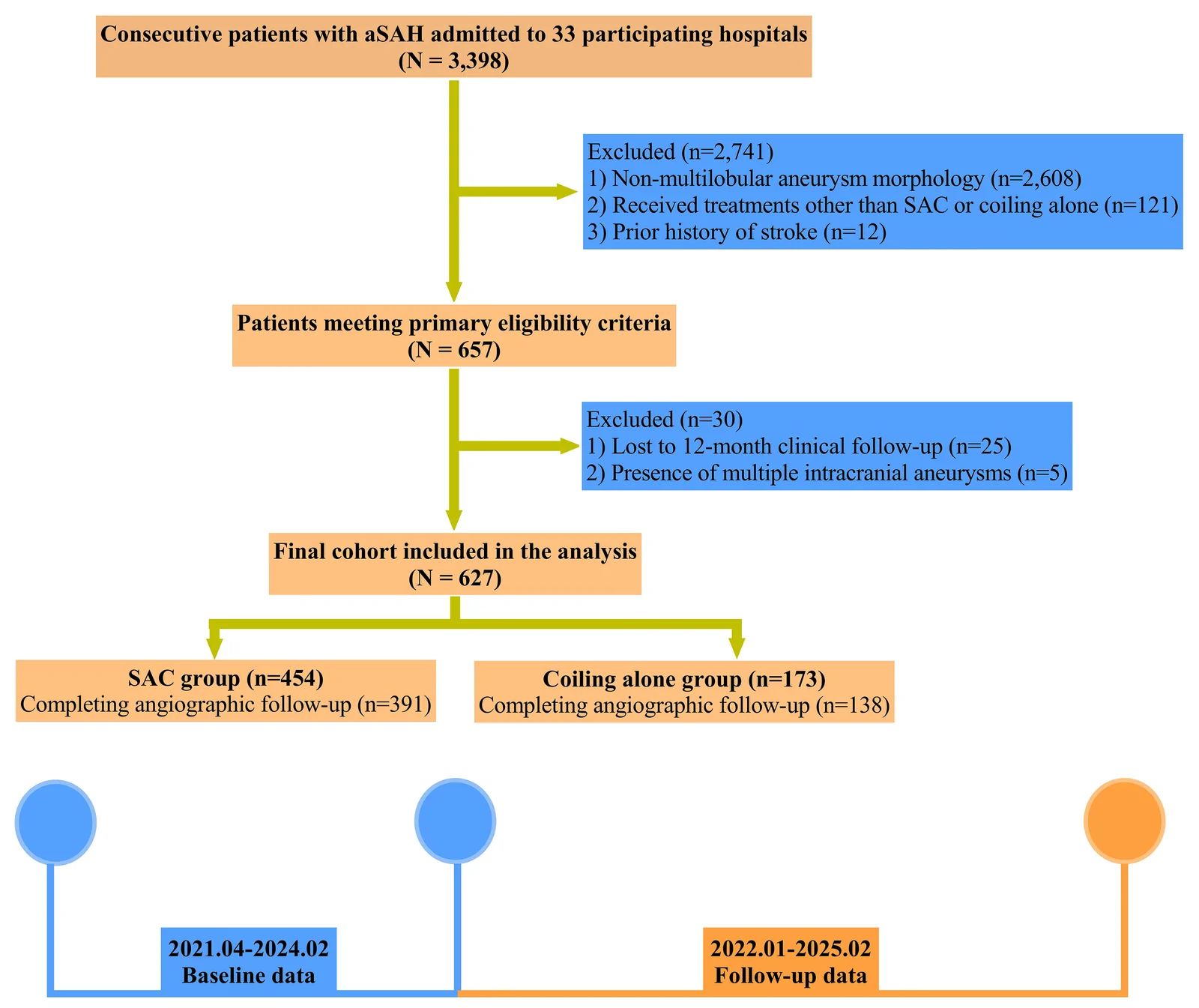
**Study flowchart.** aSAH indicates aneurysmal subarachnoid hemorrhage; and SAC, stent-assisted coiling.

### Data Collection

Detailed data for all enrolled patients were prospectively collected via standardized electronic case report forms, covering 4 core dimensions: (1) baseline demographics and clinical characteristics, including age, sex, prior vascular risk factors (smoking, alcohol consumption, hypertension, diabetes, heart disease, and dyslipidemia), vital signs on admission (temperature, heart rate, systolic blood pressure, and diastolic blood pressure), and disease severity assessments via the Hunt-Hess and modified Fisher scales; (2) aneurysm morphology and imaging features, assessed by 2 independent neuroradiologists based on preprocedural angiography, including the specific location of the culprit aneurysm, maximum diameter, neck width, dome-to-neck ratio, bifurcation involvement, and the presence of incorporated branches; (3) perioperative parameters and safety outcomes, detailing the interval from onset to treatment, total procedural duration, specific endovascular intervention, perioperative antiplatelet therapy, other neurosurgical adjunctive measures (external ventricular drainage, decompressive craniectomy, and lumbar drainage), with a focus on monitoring perioperative complications such as rerupture, rebleeding, intraprocedural thrombosis (IPT), perioperative ischemic stroke (IS), and cerebral vasospasm (CVS); (4) functional prognosis and imaging follow-up outcomes, utilizing the modified Rankin Scale (mRS) score to record neurological status at discharge and at 12 months, along with all-cause mortality (ACM). The Raymond-Roy Occlusion Classification (RROC) was applied to evaluate the degree of aneurysm packing postprocedure and during the follow-up period, as well as long-term recurrence.

Additional pretreatment anatomic variables included the arterial segment, detailed aneurysm configuration, aneurysm size category, parent-artery diameter, bifurcation involvement, and incorporated branches. Stent type and size were recorded for patients who underwent SAC. Retreatment was recorded as a binary follow-up outcome; its timing, indication, and treatment modality were not systematically retained.

### Treatment Timing and Antiplatelet Management

After admission, patients underwent emergency stabilization and diagnostic evaluation, and definitive endovascular treatment was performed as early as clinically and technically feasible during the index hospitalization. The registry did not impose a uniform mandatory interval from rupture to treatment. Treatment timing reflected the patient’s neurological and systemic condition, the need to manage hydrocephalus or raised intracranial pressure, aneurysm anatomy, referral or interhospital transfer, and the availability of the treating team. For SAC, intravenous tirofiban was administered as a 12-µg/kg loading dose over 30 minutes, followed by an infusion of 0.1 µg/(kg·min) for ≈24 hours. After postprocedural cranial computed tomography excluded new or clinically significant progressive intracranial hemorrhage, treatment was transitioned within 24 hours to aspirin 100 mg once daily plus clopidogrel 75 mg once daily. In patients with clopidogrel resistance, clopidogrel was replaced by ticagrelor (180-mg loading dose followed by 90 mg twice daily) in combination with aspirin. Dual antiplatelet therapy was generally continued for 6 months and then changed to single antiplatelet therapy. For coiling alone, routine stent-related tirofiban bridging and mandatory dual antiplatelet therapy were not prescribed. Antiplatelet or rescue antithrombotic treatment was used selectively, particularly in the presence of intraprocedural thrombus formation, distal embolization, parent-artery flow compromise, or clinically relevant coil protrusion. In both groups, the regimen and duration were adjusted according to individual hemorrhagic risk, active bleeding events, the need for external ventricular drainage or other neurosurgical procedures, and the overall clinical condition.

### Outcome Measures

The predefined outcome evaluation system in this study comprised 3 core dimensions: (1) safety outcomes, utilized as key parameters for assessing the clinical risk of different interventional modalities, which were strictly defined and monitored, primarily including rerupture, rebleeding, IPT, IS, and CVS; (2) clinical functional and survival outcomes, assessed by independent physicians not involved in the surgical intervention using the mRS score at discharge (discharge mRS score) and during the 12-month follow-up period (outcome), where mRS score of 0 to 2 was defined as a favorable prognosis and 3 to 6 as an unfavorable prognosis, with rigorous tracking of ACM during the follow-up period^14^; (3) angiographic outcomes, classified using the RROC immediately after treatment (initial RROC) and at angiographic follow-up (follow-up RROC) to characterize complete occlusion, residual neck, or residual aneurysm.^15^ Retreatment during follow-up was analyzed separately as a binary clinical-management outcome.

### Definition of RMIA

RMIA classification was based on pretreatment 3-dimensional digital subtraction angiography (DSA). A ruptured multilobulated intracranial aneurysm was defined as an aneurysm containing at least 1 distinct secondary outpouching, representing a true additional lobe or prominent daughter sac, that was separated from the primary aneurysm sac by a discernible waist or morphological inflection point. Small, shallow blebs and minor wall irregularities without an independently identifiable lobular compartment were not classified as multilobulation. All included lesions met this irregular morphological criterion; no further categorization by the number of lobules was undertaken. Pretreatment images were evaluated using the independent dual-reader and adjudication procedure described below.

### Imaging Assessment and Follow-Up

Pretreatment and follow-up examinations were independently assessed by 2 experienced radiologists holding the rank of Associate Chief Physician or above. Neither assessor participated in patient recruitment, treatment, the endovascular procedure, clinical data collection, or the conduct of this study. They were unaware of which patients were included in the analytic cohort and were not provided with clinical characteristics or clinical outcomes. Any disagreement was independently adjudicated by a third radiologist of equivalent seniority. Immediate postprocedural angiograms were evaluated independently by 2 assessors. One was the leader of the treating team, a Chief Physician in neurology with expertise in neurovascular intervention, and the other was an independent radiologist holding the rank of Associate Chief Physician or above who had not participated in the study. Disagreement was adjudicated by a third senior radiologist.

A dedicated follow-up team contacted participants and arranged scheduled return visits for angiographic assessment. DSA was the preferred follow-up modality whenever clinically feasible. Computed tomography angiography was accepted when a patient declined invasive DSA or when DSA was considered clinically or practically inappropriate. All available DSA and computed tomography angiography examinations were anonymized and submitted to the core imaging laboratory for central assessment using the same predefined criteria and dual-reader adjudication procedure. Treatment allocation was not supplied to follow-up assessors; however, complete masking of treatment modality could not always be guaranteed because coils or stents could be visible on the images.

### Statistical Analysis

Baseline characteristics were categorized into 2 groups based on the treatment modality (coiling alone versus SAC). Continuous variables were assessed for normal distribution using the Kolmogorov-Smirnov test. Normally distributed data were expressed as mean±SD, with intergroup comparisons performed using the independent-samples Student *t* test. Non-normally distributed data were presented as median and interquartile range (median [interquartile range]), and compared using the Mann-Whitney *U* test. Categorical variables were presented as frequencies and percentages (n [%]), with intergroup differences assessed using the χ^2^ test or Fisher exact test.

Given the observational design, propensity score overlap weighting was used to estimate the average treatment effect in the overlap population (ATO). The primary ATO analysis included 610 patients with complete data for the 16 prespecified pretreatment covariates. Propensity scores were estimated by logistic regression using age, sex, smoking history, alcohol consumption, hypertension, diabetes, heart disease, dyslipidemia, Hunt-Hess grade, modified Fisher scale, aneurysm location, maximum aneurysm diameter, neck width, dome-to-neck ratio, bifurcation involvement, and an incorporated branch. If e(X) denotes the estimated probability of SAC, patients in the SAC group received a weight of 1−e(X), whereas patients in the coiling-alone group received a weight of e(X). Covariate balance was assessed using absolute standardized mean differences before and after weighting, with a value <0.10 indicating adequate balance. Logistic regression–based overlap weighting can produce exact or numerically near-exact mean balance for covariates included in the propensity model, but it does not address confounding by unmeasured or incompletely measured factors. The Kish effective sample size (ESS) was calculated as (Σw)^2^/Σw^2^.

Associations between treatment modality and binary outcomes were estimated using logistic regression and reported as odds ratios (ORs) with 95% CIs. In the unweighted cohort, model 1 was unadjusted. Model 2 adjusted for age, sex, smoking history, alcohol consumption, hypertension, diabetes, dyslipidemia, heart disease, Hunt-Hess grade, and modified Fisher scale score. Model 3 additionally adjusted for aneurysm location, maximum aneurysm diameter, neck width, dome-to-neck ratio, bifurcation involvement, an incorporated branch, onset-to-treatment interval, procedure duration, external ventricular drainage, decompressive craniectomy, lumbar drainage, and perioperative antiplatelet therapy.

In the weighted cohort, the primary model included treatment modality as the sole explanatory variable and incorporated the ATO weights. A doubly robust model additionally adjusted for external ventricular drainage, decompressive craniectomy, lumbar drainage, and perioperative antiplatelet therapy, which were not included in the propensity model. Robust variance estimates were used for weighted models; center-clustered standard errors were examined in prespecified robustness analyses.

To evaluate the influence of missing angiographic outcomes, follow-up completion was compared between treatment groups, and deterministic sensitivity analyses assigned all missing outcomes to complete occlusion, all to incomplete occlusion, or to treatment-differential extreme scenarios. A stabilized inverse-probability-of-observation weighting analysis was also performed. The probability of angiographic follow-up was estimated by logistic regression including treatment modality and the 16 propensity covariates; the stabilized numerator was the observed follow-up proportion within each treatment group, and the resulting observation weight was multiplied by the ATO weight. Scenario analyses encompassing the full cohort used prespecified single imputation for the limited baseline missingness: the modal category for dyslipidemia and the median for 2 nonnumeric dome-to-neck ratio entries. Additional sensitivity analyses added parent-artery diameter to the propensity model and examined alternative handling of baseline missingness, numeric coding of propensity covariates, and reclassification of 2 records with discordant treatment documentation. Center-clustered SEs were used for these sensitivity estimates.

Exploratory ATO-weighted subgroup analyses were performed according to bifurcation involvement, aneurysm size (<10 versus ≥10 mm), and wide-neck status. Treatment-effect heterogeneity was evaluated using interaction terms and corresponding *P* values. Estimates were reported as not calculable when outcome counts were insufficient. These exploratory analyses are presented in Table S10.

All statistical inferences were 2-sided, with *P*<0.05 considered statistically significant. All statistical analyses were performed using R software (version 4.5.3).

## Results

### Baseline Characteristics and Covariate Balance

A total of 627 patients with RMIA were included, comprising 173 in the coiling-alone group and 454 in the SAC group (Table 1). Before weighting, the SAC group included a higher proportion of male patients than the coiling-alone group (68.94% versus 52.60%; *P*<0.001). Aneurysms treated with SAC had a larger mean neck width (3.88 ± 1.54 versus 3.32 ± 1.44 mm; *P*<0.001) and a lower mean dome-to-neck ratio (1.48 ± 0.81 versus 1.80 ± 0.99; *P*<0.001), and aneurysm location differed between groups (*P*=0.002). Procedure duration was longer in the SAC group (146.91 ± 59.18 versus 127.38 ± 60.72 minutes; *P*<0.001), and dual antiplatelet therapy was more frequent (89.87% versus 55.49%; *P*<0.001). In the unweighted cohort, perioperative IS (7.05% versus 2.31%; *P*=0.023) and IPT (4.41% versus 1.16%; *P*=0.048) were more frequent in the SAC group, whereas rerupture, rebleeding, CVS, functional outcomes, and ACM did not differ significantly. Initial RROC class I occlusion (66.52% versus 56.07%; *P*<0.001) and follow-up RROC class I occlusion (75.19% versus 55.07%; *P*<0.001) were also more frequent in the SAC group (Figure 3).

**Table 1.**
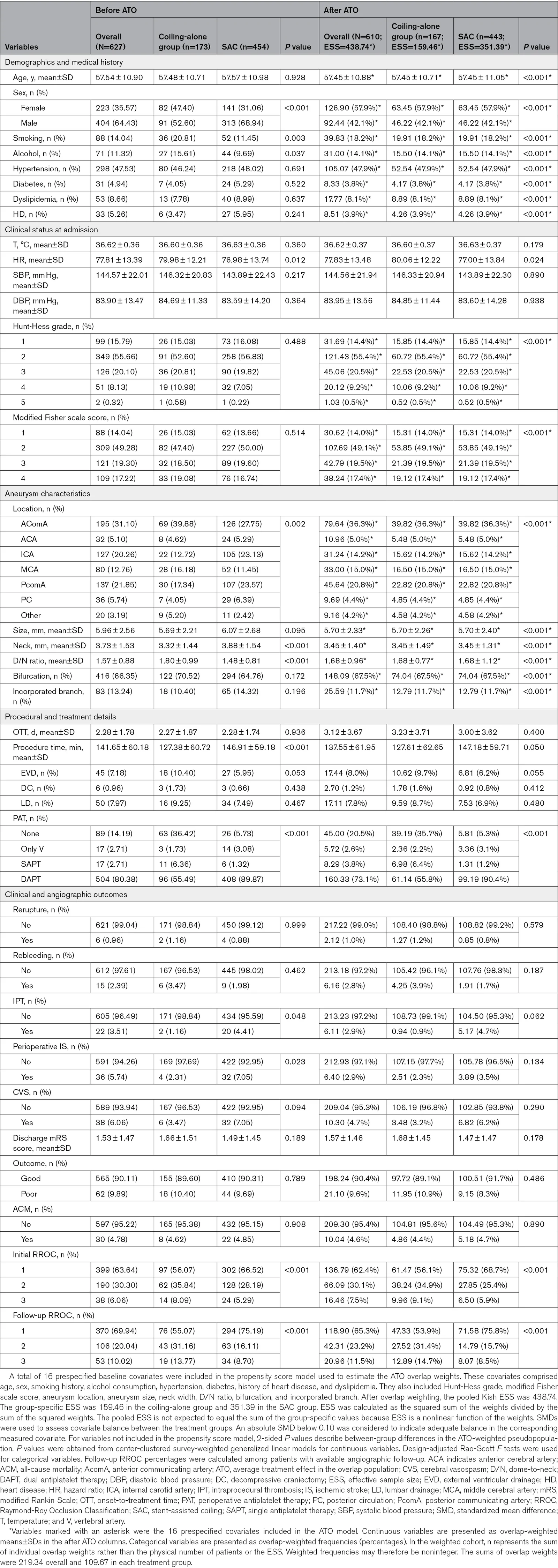
Baseline Characteristics

**Figure 3.**
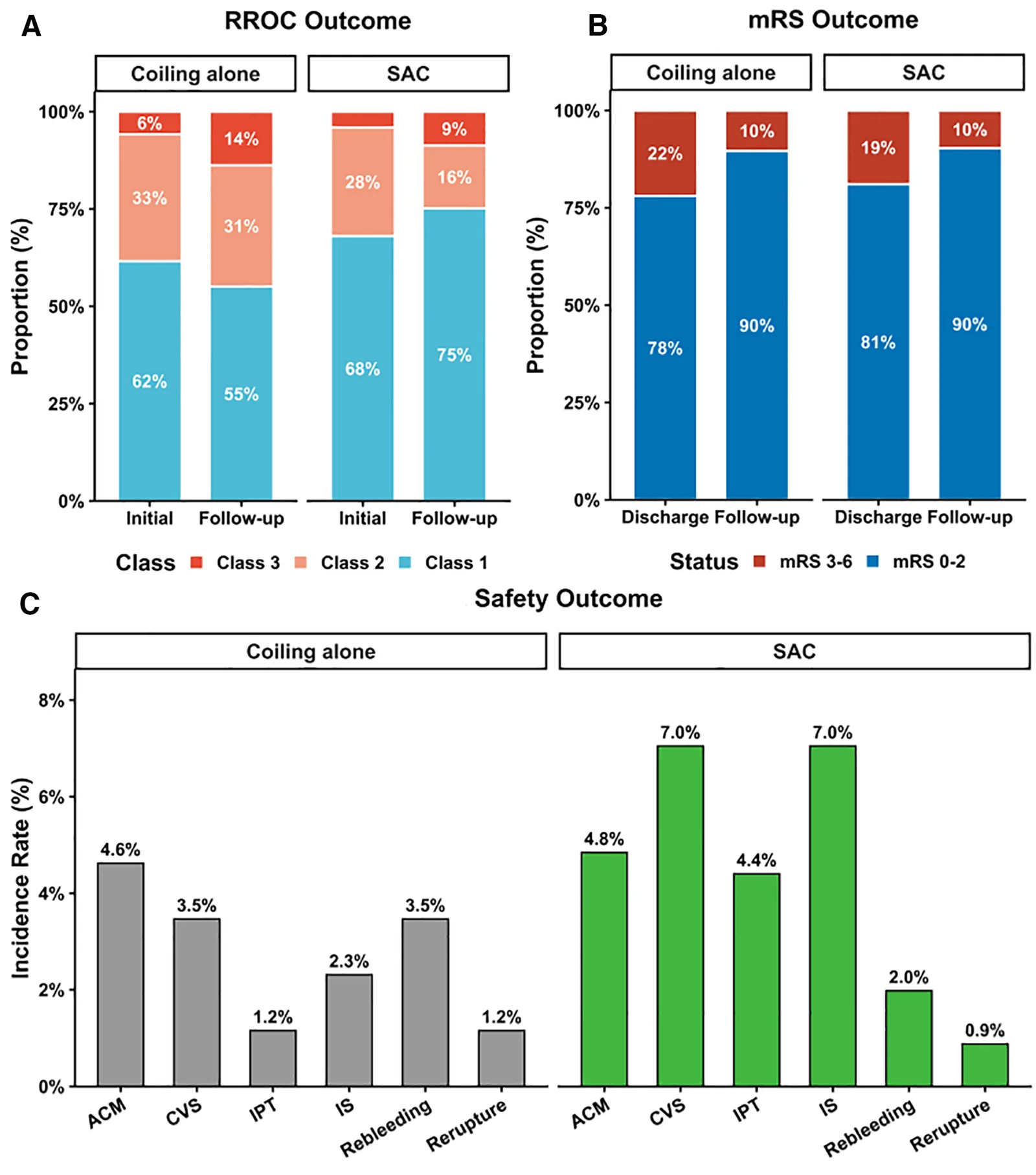
**Stacked bar charts.** Comparing the primary clinical, angiographic, and safety outcomes between the coiling alone and stent-assisted coiling (SAC) groups. **A**, Distribution of Raymond-Roy Occlusion Classification (RROC) grades at initial postprocedure and long-term follow-up, demonstrating a significantly higher proportion of complete occlusion (class I) in the SAC group at both time points. **B**, Distribution of functional status, categorized by modified Rankin Scale (mRS) scores (0–2 vs 3–6), at hospital discharge and 12-month follow-up, indicating comparable functional outcomes between the 2 treatment modalities. **C**, Incidence of major perioperative and long-term safety end points, highlighting that SAC does not significantly increase the overall risk of hemorrhagic or hemic complications compared with coiling alone. ACM indicates all-cause mortality; CVS, cerebral vasospasm; IPT, intraprocedural thrombosis; and IS, ischemic stroke.

The primary ATO analysis included 610 patients with complete propensity model covariates, including 167 treated with coiling alone and 443 treated with SAC. The pooled Kish ESS was 438.74; the group-specific ESS values were 159.46 for coiling alone and 351.39 for SAC (Table 1). Before weighting, the largest absolute standardized mean differences were observed for neck width (0.364), dome-to-neck ratio (0.346), and sex (0.335). After ATO weighting, all 16 modeled covariates had absolute standardized mean differences of 0.000 to 3 decimal places, below the prespecified 0.10 threshold (Figure 4).

**Figure 4.**
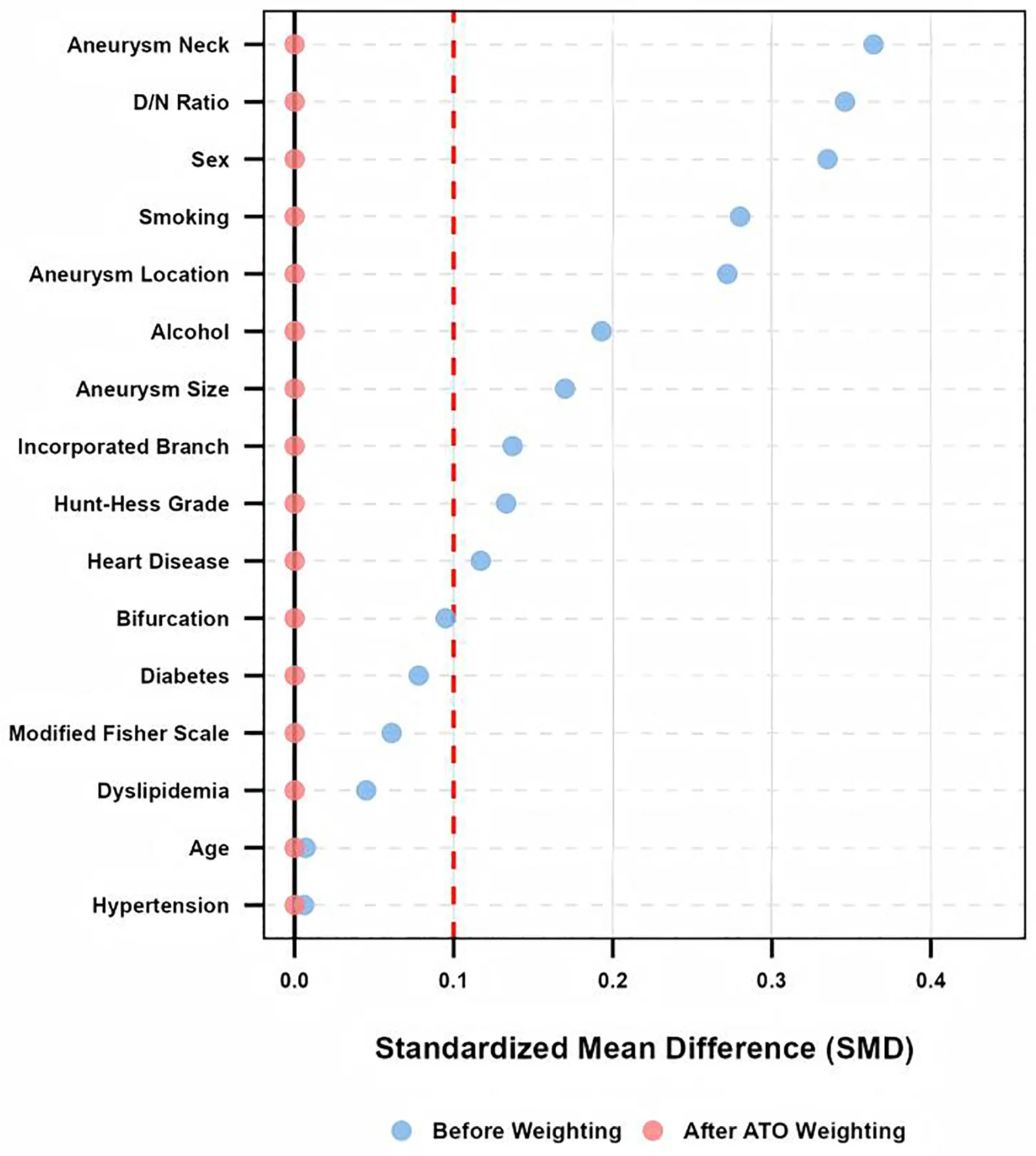
**Covariate balance before and after overlap weighting.** Love plot showing the absolute standardized mean differences (SMDs) for the 16 prespecified pretreatment covariates before and after overlap weighting (average treatment effect in the overlap population [ATO]). The vertical dashed line at an absolute SMD of 0.10 represents the prespecified threshold for adequate covariate balance. After ATO weighting, the absolute SMDs for all 16 covariates, including sex, aneurysm location, neck width, and dome-to-neck ratio, were <0.10 and were 0.000 to 3 decimal places, indicating adequate numerical balance in the measured pretreatment covariates between the coiling-alone and stent-assisted coiling groups. D/N indicates dome-to-neck.

In the ATO-weighted descriptive analysis, perioperative IS occurred in 3.5% of the SAC group and 2.3% of the coiling-alone group (*P*=0.134), whereas IPT occurred in 4.7% and 0.9%, respectively (*P*=0.062). No statistically significant weighted between-group differences were observed for rerupture, rebleeding, CVS, discharge mRS score, follow-up functional outcome, or ACM. The weighted RROC distributions differed at both the initial and follow-up assessments (both *P*<0.001). Initial RROC class I occlusion was observed in 68.7% of the SAC group and 56.1% of the coiling-alone group, and follow-up RROC class I occlusion was observed in 75.8% and 53.9%, respectively (Table 1).

### Regression Analyses

In the weighted unadjusted models, SAC was not significantly associated with rerupture (OR, 0.666 [95% CI, 0.147–3.005]; *P*=0.582), rebleeding (OR, 0.439 [95% CI, 0.121–1.589]; *P*=0.198), IPT (OR, 5.721 [95% CI, 0.673–10.349]; *P*=0.102), perioperative IS (OR, 1.574 [95% CI, 0.659–2.870]; *P*=0.063), or CVS (OR, 2.025 [95% CI, 0.513–7.988]; *P*=0.299; Table 2). No significant associations were identified for poor functional outcome at discharge (OR, 0.833 [95% CI, 0.499–1.391]; *P*=0.468), poor functional outcome at follow-up (OR, 0.745 [95% CI, 0.315–1.764]; *P*=0.487), or ACM (OR, 1.070 [95% CI, 0.394–2.906]; *P*=0.890). The doubly robust models likewise showed no statistically significant associations for the evaluated safety or clinical outcomes (all *P*≥0.071).

**Table 2.**
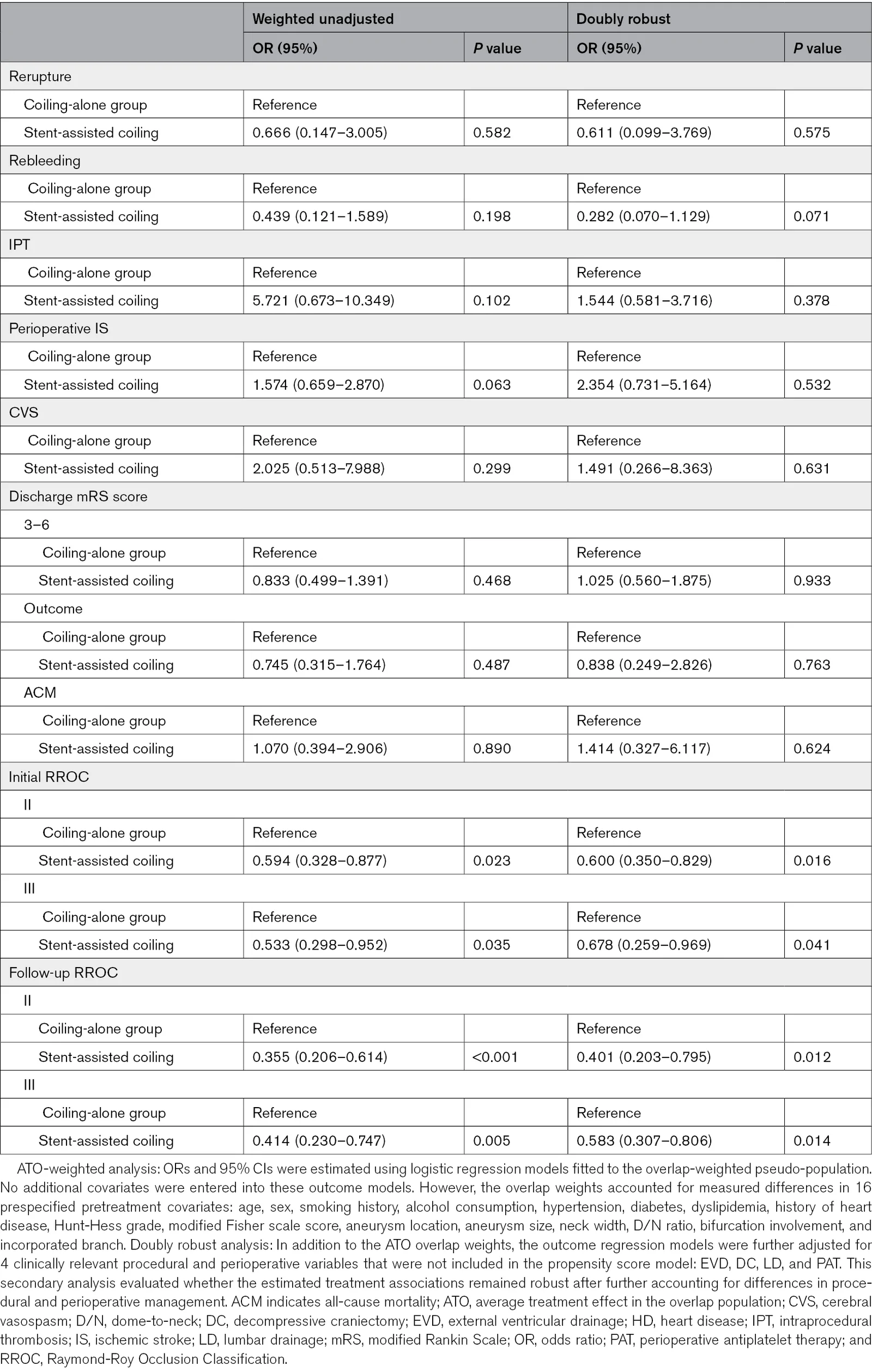
Logistic Regression Analyses of Clinical and Angiographic Outcomes After ATO Overlap Weighting

SAC was associated with lower odds of incomplete angiographic occlusion. In the weighted unadjusted analysis, the ORs were 0.594 (95% CI, 0.328–0.877; *P*=0.023) for initial RROC class II versus I and 0.533 (95% CI, 0.298–0.952; *P*=0.035) for initial RROC class III versus I. At follow-up, the corresponding ORs were 0.355 (95% CI, 0.206–0.614; *P*<0.001) and 0.414 (95% CI, 0.230–0.747; *P*=0.005). These associations remained statistically significant in the doubly robust models for initial RROC class II versus I (OR, 0.600 [95% CI, 0.350–0.829]; *P*=0.016), initial RROC class III versus I (OR, 0.678 [95% CI, 0.259–0.969]; *P*=0.041), follow-up RROC class II versus I (OR, 0.401 [95% CI, 0.203–0.795]; *P*=0.012), and follow-up RROC class III versus I (OR, 0.583 [95% CI, 0.307–0.806]; *P*=0.014; Table 2).

### Sensitivity and Additional Analyses

The fully adjusted unweighted analyses yielded similar angiographic findings, with ORs of 0.556 ([95% CI, 0.335–0.922]; *P*=0.023) and 0.605 ([95% CI, 0.192–0.945]; *P*=0.031) for initial RROC classes II and III versus I, respectively, and 0.345 ([95% CI, 0.184–0.647]; *P*<0.001) and 0.381 ([95% CI, 0.158–0.914]; *P*=0.031) for follow-up RROC classes II and III versus I, respectively (Table S1). Detailed weight diagnostics confirmed the pooled and group-specific ESS values (Table S2), and numerical balance diagnostics confirmed an absolute post-ATO standardized mean difference of 0.000 for each modeled covariate to 3 decimal places (Table S3).

Angiographic follow-up was available for 529 of 627 patients (84.4%). Completion was 86.1% in the SAC group and 79.8% in the coiling-alone group (*P*=0.064; Table S4). In the complete-case analysis, SAC was associated with lower odds of follow-up incomplete occlusion (OR, 0.366 [95% CI, 0.238–0.562]; *P*<0.001). The association persisted when all missing outcomes were assigned either complete occlusion (OR, 0.452 [95% CI, 0.303–0.673]; *P*<0.001) or incomplete occlusion (OR, 0.386 [95% CI, 0.254–0.587]; *P*<0.001), and after stabilized inverse-probability-of-observation weighting (OR, 0.354 [95% CI, 0.236–0.533]; *P*<0.001). In the deliberately adverse treatment-differential scenario, in which all missing SAC outcomes were assigned incomplete occlusion and all missing coiling-alone outcomes were assigned complete occlusion, the association was attenuated to the null (OR, 0.909 [95% CI, 0.527–1.567]; *P*=0.721; Table S5).

Additional adjustment for parent-artery diameter produced similar estimates for follow-up incomplete occlusion (OR, 0.369 [95% CI, 0.235–0.580]; *P*<0.001), RROC class II versus I (OR, 0.345 [95% CI, 0.195–0.610]; *P*<0.001), and RROC class III versus I (OR, 0.421 [95% CI, 0.241–0.738]; *P*=0.004). Alternative handling of baseline missingness, numeric coding of propensity covariates, and reclassification of 2 discordant treatment records did not materially alter the angiographic estimates; the evaluated safety and functional outcomes remained nonsignificant (Table S6).

Retreatment occurred in 27 of 454 patients treated with SAC (5.9%) and 19 of 173 treated with coiling alone (11.0%; *P*=0.039; Table S7). The median onset-to-treatment interval was 2 days (interquartile range, 1–4 days); 44.5% of patients were treated within 1 day, 74.5% within 3 days, and 91.7% within 7 days (Table S8). The source counts and denominators used to verify the distributions displayed in Figure 3 are provided in Table S9.

### Exploratory Subgroup Analyses

Exploratory subgroup analyses are presented in Table S10. No statistically significant treatment interactions were identified for bifurcation status or aneurysm size in estimable comparisons. Wide-neck status modified the association for initial RROC class II versus I (*P*_interaction_=0.040) and follow-up RROC class III versus I (*P*_interaction_=0.006). For follow-up RROC class III versus I, the OR was 0.304 ([95% CI, 0.159–0.582]; *P*<0.001) in the wide-neck subgroup and 1.220 ([95% CI, 0.486–3.060]; *P*=0.647) in the non-wide-neck subgroup. No statistically significant interactions were observed for the evaluable functional or safety outcomes. Several estimates were not calculable because of sparse or empty outcome cells.

## Discussion

The SANE trial represents one of the most extensive data sets to date comparing SAC with coiling alone in patients with aSAH caused by RMIA. Through a consecutive analysis of 627 patients, the study systematically evaluated differences in perioperative safety, functional outcomes, and angiographic durability between the 2 strategies. Regarding perioperative safety, crude data showed higher rates of IPT and perioperative IS in the SAC group; however, neither association remained statistically significant after ATO weighting or doubly robust adjustment. No statistically significant between-group differences were identified in rerupture, rebleeding, or CVS. Functional outcomes were also similar, with no detectable differences in poor functional status at discharge or follow-up, or in ACM. In the angiographic evaluation, SAC was associated with more favorable initial and long-term occlusion. In the doubly robust analysis, the odds of follow-up RROC class II versus I and class III versus I were lower after SAC, with ORs of 0.401 and 0.583, respectively (Table 2). This angiographic advantage was not accompanied by a detectable functional or survival benefit during the available follow-up.

The occurrence and rupture of intracranial aneurysms result from complex interactions between abnormal hemodynamic stimuli and degenerative changes in the local vessel-wall microenvironment.^16,17^ Hemodynamic stress is central to this process. Sustained high wall shear stress (WSS) and an elevated oscillatory shear index may promote endothelial dysfunction and trigger chronic inflammatory cascades, including macrophage recruitment and increased expression of matrix metalloproteinases.^18,19^ These processes disrupt the internal elastic lamina, promote apoptosis of medial smooth-muscle cells, and progressively degrade the ECM (extracellular matrix), ultimately reducing the load-bearing capacity of the vessel wall.^20,21^ RMIA morphology may represent a macroscopic manifestation of the spatial heterogeneity of these pathological processes. High-velocity impingement flow may produce locally elevated WSS, whereas deep regions of the sac and spaces within individual lobes may be exposed to low and highly oscillatory WSS.^21^ Flow stasis in these low-WSS regions may promote microthrombosis, wall ischemia or hypoxia, and further inflammatory degeneration.^22^ The coexistence of high- and low-WSS regions, together with complex wall-stress concentration, may increase susceptibility to focal wall injury and rupture.^23^ This morphological and hemodynamic complexity may also contribute to nonuniform coil distribution, incomplete occlusion, and recurrence after coiling alone.

With continuing advances in endovascular devices, treatment strategies for intracranial aneurysms have expanded from coiling alone to adjunctive techniques, including SAC, dual-microcatheter and balloon-assisted coiling, flow diversion, and other intraluminal devices.^24,25^ In acutely ruptured aneurysms, coiling alone and SAC remain 2 widely used strategies, although treatment selection is constrained by aneurysm anatomy, the need for antiplatelet therapy, and the patient’s clinical condition.^26^ SAC entails greater procedural and perioperative complexity, and previous studies have reported both thromboembolic and hemorrhagic concerns.^7,27^ In carefully selected patients, intraoperative tirofiban bridging followed by standard postoperative dual antiplatelet therapy may help control device-related thrombotic risk, but the hemorrhagic risk must be assessed throughout treatment.^28,29^ Mechanically, a stent provides neck scaffolding that may prevent coil protrusion, improve coil stability, and support durable aneurysm occlusion.^30–33^ These effects provide a plausible explanation for the more durable angiographic occlusion observed after SAC in the present cohort.

Despite these morphological advantages, caution remains warranted regarding thromboembolic risk during the acute phase of SAC. The implanted metallic surface is thrombogenic, and the systemic and local prothrombotic states associated with aSAH may further increase the risk of acute in-stent thrombosis and distal embolization.^34,35^ This concern complicates emergency antiplatelet management because thrombosis prevention must be balanced against rerupture and procedure-related bleeding. Coiling alone can secure the rupture site without permanent intraluminal metal and usually requires less intensive antiplatelet management, which may be advantageous in patients with a high hemorrhagic risk.^36,37^ However, without intraluminal mechanical support, coil stability and durable neck coverage may be more difficult to achieve in anatomically complex lesions. Persistent or recurrent filling may subsequently develop under continued arterial-flow impingement.^38,39^ Previous studies have reported angiographic recanalization or recurrence rates of ≈20% to 33% after coiling alone, particularly in aneurysms with residual necks or complex morphology.^38,39^ In RMIA, overall 3-dimensional complexity should therefore be considered together with absolute neck size. In the present subgroup analysis, wide-neck status modified the association for selected RROC comparisons, whereas no statistically significant interaction was identified for the evaluable functional or safety outcomes. Treatment selection should consequently integrate 3-dimensional anatomy, coil stability, hemorrhagic risk, and clinical condition rather than rely on lobular complexity alone.

Compared with saccular aneurysms with regular anatomy, multilobulated lesions present greater technical challenges during endovascular treatment. He et al^40^ reported that irregular geometry may produce marked intra-aneurysmal flow disturbance, nonuniform coil distribution, and subsequent coil compaction, and identified lobulation as a predictor of long-term angiographic recurrence. High-quality prospective cohorts or large multicenter studies focused specifically on RMIA remain limited. Previous evidence has largely been derived from subgroup analyses or small case series, with restricted sample sizes and limited adjustment for treatment-selection factors related to anatomic complexity. In the present study, ATO-weighted and doubly robust analyses consistently associated SAC with lower odds of incomplete occlusion. For follow-up RROC class II versus I and class III versus I, the doubly robust ORs were 0.401 and 0.583, respectively (Table 2), and the overall distributions are shown in Figure 3A. Retreatment was also recorded less frequently after SAC than after coiling alone (5.9% versus 11.0%). Nevertheless, functional outcome and mortality did not differ detectably between the groups. Thus, the observed angiographic durability should be interpreted as a clinically relevant anatomic benefit, rather than evidence of functional or survival superiority.

Methodologically, this study used a multicenter prospective registry and prespecified ATO and doubly robust models to compare the treatment groups within an observational framework. ATO achieved numerical balance across the 16 measured pretreatment covariates, while additional analyses incorporating parent-artery diameter and center-level clustering produced consistent estimates. These approaches strengthen comparability with respect to the measured factors and provide a transparent assessment of the association between treatment strategy and outcome. Taken together, the findings support an association between SAC and improved angiographic durability in RMIA, while treatment choice remains individualized rather than determined by a single anatomic feature.

Several limitations should be acknowledged. First, this post hoc observational analysis was not randomized, and residual confounding may persist despite ATO and doubly robust adjustment. Treatment selection remained influenced by operator judgment, incompletely measured 3-dimensional anatomy, embolization end points, and center-level practice; stent type and coil packing density were treatment-specific or postselection features rather than pretreatment covariates. Second, the independently assessed operational definition of RMIA has not been externally validated. Third, angiographic follow-up was unavailable for 15.6% of patients, and the adverse treatment-differential sensitivity scenario attenuated the angiographic association to the null. The use of both DSA and computed tomography angiography and the visibility of treatment devices may also have introduced measurement heterogeneity and limited complete masking. Finally, retreatment was recorded only as a binary outcome without detailed timing, indication, or technique, and sparse subgroup estimates require cautious interpretation. These findings should therefore be interpreted as associations and validated in further prospective studies.

## Conclusions

In this multicenter observational cohort of RMIA, SAC was associated with more durable initial and follow-up angiographic occlusion than coiling alone, whereas the evaluated perioperative safety, functional, and survival outcomes did not differ detectably. Treatment should remain individualized, and coiling alone remains appropriate for selected anatomically favorable lesions in which stable occlusion can be achieved without adjunctive stenting.

## ARTICLE INFORMATION

### Acknowledgments

The authors thank the neurovascular centers of the 33 collaborative hospitals for providing clinical cases, which formed the essential foundation for this study. Furthermore, the authors acknowledge the efforts of all team members responsible for data collection, verification, and organization, as their meticulous work ensured the integrity and reliability of the study data.

### Author Contributions

Drs Xiao and Liu were responsible for the conception and design of the study. Drs Mi and Duan oversaw data acquisition and patient eligibility screening. Dr Xiao performed the data analysis and interpretation, conducted the statistical analysis, and drafted the manuscript. Drs Liu, Wang, and Duan critically revised the manuscript for important intellectual content and provided study supervision and project administration. Drs Duan and Liu were responsible for funding acquisition. All authors have read and approved the final version of the manuscript for submission.

### Disclosures

None.

### Supplemental Material

Tables S1–S10

STROBE Checklist

## Supplementary Material


