# Supplementary material for: Efficacy and Safety of Stent-Assisted Coiling Versus Coiling Alone for Ruptured Multilobed Aneurysms: A Multicenter Study
Source: Stroke Vasc Interv Neurol. 2026 Sep 3;6(6):e002556. doi: 10.1161/SVIN.126.002556 (PMC13727198; doi:10.1161/SVIN.126.002556)
Supplement: Supplementary file 1 [file svi2-6-e002556-s002.pdf]

SUPPLEMENTAL MATERIALS

Efficacy and Safety of Stent-Assisted Coiling vs Coiling Alone for Ruptured Multi-Lobed Aneurysms: A Multicenter Study

ZhenKun Xiao[1#], PhD, ShanShan Mi[1#], MD, Bing Wang[1], PhD, YongHong Duan[1*], MD, and AiHua Liu[1,2,3*], PhD

[1]Hengyang Key Laboratory of Hemorrhagic Cerebrovascular Disease, Department of Neurosurgery, the Second Affiliated Hospital, Hengyang Medical School, University of South China, Hengyang, Hunan, China
[2]Beijing Neurosurgical Institute, Capital Medical University, Beijing, China
[3]Department of Interventional Neuroradiology, Beijing Tiantan Hospital, Beijing, China

[*]Correspondence

AiHua Liu
Department of Interventional Neuroradiology, Beijing Tiantan Hospital, Beijing, China
E-mail: 16673498449@163.com
YongHong Duan
Hengyang Key Laboratory of Hemorrhagic Cerebrovascular Disease, Department of Neurosurgery, the Second Affiliated Hospital, Hengyang Medical School, University of South China, Hengyang 421000, Hunan, China
E-mail: 13875643396@163.com

[#]Co-first authors
The first two authors contributed equally to this work.

Supplementary Table S1. Multivariable Logistical Regression Analysis before ATO

		Model 1		Model 2		Model 3	
		**OR (95%)**	***P***	**OR (95%)**	***P***	**OR (95%)**	***P***
**Rerupture**	Coiling alone group	Reference		Reference		Reference	
	Stent-assisted coiling	0.760 (0.138-4.187)	0.753	0.350 (0.055-2.234)	0.267	0.156 (0.002-13.853)	0.417
**Rebleeding**	Coiling alone group	Reference		Reference		Reference	
	Stent-assisted coiling	0.563 (0.197-1.606)	0.283	0.435 (0.143-1.322)	0.142	0.203 (0.032-1.296)	0.092
**IPT**	Coiling alone group	Reference		Reference		Reference	
	Stent-assisted coiling	3.940 (0.911-17.039)	0.066	5.194 (1.117-24.140)	0.036	4.703 (0.545-40.569)	0.159
**Perioperative IS**	Coiling alone group	Reference		Reference		Reference	
	Stent-assisted coiling	3.204 (1.116-9.198)	0.030	2.606 (0.883-7.693)	0.083	1.607 (0.480-5.386)	0.442
**CVS**	Coiling alone group	Reference		Reference		Reference	
	Stent-assisted coiling	2.111 (0.867-5.140)	0.100	2.120 (0.853-5.268)	0.106	1.412 (0.463-4.306)	0.544
**Discharge mRS**							
**3-6**	Coiling alone group	Reference		Reference		Reference	
	Stent-assisted coiling	0.830 (0.540-1.276)	0.396	0.861 (0.513-1.445)	0.572	1.029 (0.503-2.104)	0.938
**Outcome**	Coiling alone group	Reference		Reference		Reference	
	Stent-assisted coiling	0.924 (0.518-1.648)	0.789	0.895 (0.461-1.740)	0.744	1.006 (0.370-2.738)	0.991
**ACM**	Coiling alone group	Reference		Reference		Reference	
	Stent-assisted coiling	1.050 (0.459-2.406)	0.908	1.175 (0.453-3.044)	0.740	1.927 (0.388-9.568)	0.422
**Initial RROC**							
**II**	Coiling alone group	Reference		Reference		Reference	
	Stent-assisted coiling	0.663 (0.454-0.970)	0.034	0.649 (0.436-0.965)	0.033	0.556 (0.335-0.922)	0.023
**III**	Coiling alone group	Reference		Reference		Reference	
	Stent-assisted coiling	0.551 (0.274-0.907)	0.016	0.673 (0.318-0.926)	0.025	0.605 (0.192-0.945)	0.031
**Follow-up RROC**							
**II**	Coiling alone group	Reference		Reference		Reference	
	Stent-assisted coiling	0.379 (0.238-0.601)	<0.001	0.360 (0.222-0.585)	<0.001	0.345 (0.184-0.647)	<0.001
**III**	Coiling alone group	Reference		Reference		Reference	
	Stent-assisted coiling	0.463 (0.250-0.856)	0.014	0.499 (0.262-0.950)	0.034	0.381 (0.158-0.914)	0.031

Model 1 no adjusted;
Model 2 adjusted for Age, Sex, Smoking, Alcohol, Hypertension, Diabetes, Dyslipidemia, HD, Hunt-Hess grade, and mFisher scale;
Model 3 adjusted for Model 2+Location, Size, Neck, D/N Ratio, Bifurcation, Incorporated Branch, OTT, Procedure Time, EVD, DC, LD, and PAT.

Supplementary Table S2. Overlap-Weight and Effective Sample Size Diagnostics

Scope	N	Sum of weights	Kish ESS	Minimum weight	Median weight	Maximum weight
Overall	610	219.34	438.74	0.041	0.288	0.935
Coiling alone	167	109.67	159.46	0.067	0.658	0.935
SAC	443	109.67	351.39	0.041	0.220	0.834

Kish ESS was calculated as (sum of weights)^2 divided by the sum of squared weights. The sum of weights is scale-dependent and is not an effective sample size. The previously reported value of 62.88 could not be reproduced as a standard ESS from the supplied data and code.

Supplementary Table S3. Numerical Standardized Mean Differences

Covariate	Absolute SMD before weighting	Absolute SMD after ATO weighting	SMD <0.10 after weighting
Age	0.007	0.000	Yes
Sex	0.335	0.000	Yes
Smoking	0.280	0.000	Yes
Alcohol	0.193	0.000	Yes
Hypertension	0.006	0.000	Yes
Diabetes	0.078	0.000	Yes
HD	0.117	0.000	Yes
Dyslipidemia	0.045	0.000	Yes
HHGrade	0.133	0.000	Yes
Fisher	0.061	0.000	Yes
Location	0.272	0.000	Yes
Size	0.170	0.000	Yes
Neck	0.364	0.000	Yes
DNRatio	0.346	0.000	Yes
Bifurcation	0.095	0.000	Yes
IncorporatedBranch	0.137	0.000	Yes

For multicategory covariates, the largest absolute level-specific SMD is shown. The corresponding graphical presentation is provided in the corrected balance plot.

Supplementary Table S4. Angiographic Follow-up Completion

Group	Total N	Angiographic follow-up	Missing angiographic follow-up	Completion rate	Fisher exact P
Coiling alone	173	138	35	79.8%	0.064
SAC	454	391	63	86.1%	0.064

Angiographic follow-up was available for 529 of 627 patients (84.4%).

Supplementary Table S5. Sensitivity Analyses for Missing Angiographic Follow-up

Analysis	Outcome	N	Events	OR (95% CI)	P
Complete-case observed follow-up	Follow-up incomplete occlusion	529	159	0.366 (0.238-0.562)	<0.001
All missing assigned complete	Follow-up incomplete occlusion	627	159	0.452 (0.303-0.673)	<0.001
All missing assigned incomplete	Follow-up incomplete occlusion	627	257	0.386 (0.254-0.587)	<0.001
SAC missing incomplete; coiling missing complete	Follow-up incomplete occlusion	627	222	0.909 (0.527-1.567)	0.721
SAC missing complete; coiling missing incomplete	Follow-up incomplete occlusion	627	194	0.192 (0.123-0.300)	<0.001
ATO x stabilized IPOW	Follow-up incomplete occlusion	529	159	0.354 (0.236-0.533)	<0.001
ATO x stabilized IPOW	Follow-up RROC II vs I	476	106	0.350 (0.205-0.598)	<0.001
ATO x stabilized IPOW	Follow-up RROC III vs I	423	53	0.362 (0.207-0.633)	<0.001

Deterministic scenarios were applied to all 627 patients using the prespecified baseline-imputation sensitivity weights. The deliberately treatment-differential worst-case scenario (all missing SAC outcomes incomplete and all missing coiling outcomes complete) is an extreme bound and attenuated the association. IPOW indicates inverse probability of observation weighting.

Supplementary Table S6. Robustness Analyses

Sensitivity analysis	Outcome	N	Events	OR (95% CI)	P
Additional parent-artery diameter in PS model	Follow-up incomplete occlusion	513	156	0.369 (0.235-0.580)	<0.001
Additional parent-artery diameter in PS model	Follow-up RROC II vs I	460	103	0.345 (0.195-0.610)	<0.001
Additional parent-artery diameter in PS model	Follow-up RROC III vs I	410	53	0.421 (0.241-0.738)	0.004
Additional parent-artery diameter in PS model	Discharge mRS 3-6	608	119	0.835 (0.496-1.406)	0.382
Additional parent-artery diameter in PS model	Follow-up mRS 3-6	608	59	0.755 (0.313-1.822)	0.516
Additional parent-artery diameter in PS model	All-cause mortality	608	28	1.080 (0.399-2.928)	0.874
Baseline mode/median imputation	IPT	627	22	6.635 (0.463-15.343)	0.641
Baseline mode/median imputation	Perioperative IS	627	36	3.649 (0.526-7.725)	0.525
Baseline mode/median imputation	Discharge mRS 3-6	627	124	0.873 (0.526-1.449)	0.586
Baseline mode/median imputation	Follow-up mRS 3-6	627	62	0.745 (0.317-1.751)	0.485
Baseline mode/median imputation	Follow-up incomplete occlusion	529	159	0.366 (0.238-0.562)	<0.001
Baseline mode/median imputation	Follow-up RROC II vs I	476	106	0.347 (0.203-0.592)	<0.001
Baseline mode/median imputation	Follow-up RROC III vs I	423	53	0.407 (0.228-0.725)	0.004
Numeric PS-covariate coding	IPT	610	21	4.809 (0.445-9.010)	0.313
Numeric PS-covariate coding	Perioperative IS	610	34	3.010 (0.313-6.900)	0.211
Numeric PS-covariate coding	Follow-up incomplete occlusion	515	157	0.388 (0.250-0.603)	<0.001
Numeric PS-covariate coding	Follow-up RROC II vs I	462	104	0.365 (0.213-0.625)	<0.001
Numeric PS-covariate coding	Follow-up RROC III vs I	411	53	0.437 (0.243-0.785)	0.008
Reclassification of two discordant treatment records	IPT	610	21	5.760 (0.798-11.451)	0.715
Reclassification of two discordant treatment records	Perioperative IS	610	34	2.548 (0.185-5.984)	0.103
Reclassification of two discordant treatment records	Follow-up incomplete occlusion	515	157	0.367 (0.224-0.603)	<0.001
Reclassification of two discordant treatment records	Follow-up RROC II vs I	462	104	0.360 (0.203-0.637)	0.001
Reclassification of two discordant treatment records	Follow-up RROC III vs I	411	53	0.383 (0.196-0.750)	0.007

All estimates compare SAC with coiling alone and use center-clustered standard errors. The two discordant records were retained according to the core analysis coding in the primary analysis; the alternative classification did not materially change the principal findings.

Supplementary Table S7. Retreatment Rates

Group	Retreatment events	Total N	Retreatment rate	Fisher exact P
Coiling alone	19	173	11.0%	0.039
SAC	27	454	5.9%	0.039

The registry retained whether retreatment occurred but did not systematically retain the indication, timing, or retreatment modality.

Supplementary Table S8. Treatment Timeline After Rupture

Group	N	Median days (IQR)	Within 1 day, n (%)	Within 3 days, n (%)	Within 7 days, n (%)
Overall	627	2 (1-4)	279 (44.5%)	467 (74.5%)	575 (91.7%)
Coiling alone	173	2 (1-4)	78 (45.1%)	129 (74.6%)	156 (90.2%)
SAC	454	2 (1-4)	201 (44.3%)	338 (74.4%)	419 (92.3%)

The onset-to-treatment interval was recorded in days; intervals shorter than 24 hours were coded as 1 day in the source registry.

Supplementary Table S9. Source Values Used to Verify Figure 3

Panel	Treatment group	Category	n	Denominator	Percentage
Initial RROC	Coiling alone	1	97	173	56.1%
Initial RROC	Coiling alone	2	62	173	35.8%
Initial RROC	Coiling alone	3	14	173	8.1%
Initial RROC	SAC	1	302	454	66.5%
Initial RROC	SAC	2	128	454	28.2%
Initial RROC	SAC	3	24	454	5.3%
Follow-up RROC	Coiling alone	1	76	138	55.1%
Follow-up RROC	Coiling alone	2	43	138	31.2%
Follow-up RROC	Coiling alone	3	19	138	13.8%
Follow-up RROC	SAC	1	294	391	75.2%
Follow-up RROC	SAC	2	63	391	16.1%
Follow-up RROC	SAC	3	34	391	8.7%
Discharge mRS 0-2 vs 3-6	Coiling alone	0	135	173	78.0%
Discharge mRS 0-2 vs 3-6	Coiling alone	1	38	173	22.0%
Discharge mRS 0-2 vs 3-6	SAC	0	368	454	81.1%
Discharge mRS 0-2 vs 3-6	SAC	1	86	454	18.9%
Follow-up mRS 0-2 vs 3-6	Coiling alone	0	155	173	89.6%
Follow-up mRS 0-2 vs 3-6	Coiling alone	1	18	173	10.4%
Follow-up mRS 0-2 vs 3-6	SAC	0	410	454	90.3%
Follow-up mRS 0-2 vs 3-6	SAC	1	44	454	9.7%
Safety outcomes	Coiling alone	ACM	8	173	4.6%
Safety outcomes	SAC	ACM	22	454	4.8%
Safety outcomes	Coiling alone	CVS	6	173	3.5%
Safety outcomes	SAC	CVS	32	454	7.0%
Safety outcomes	Coiling alone	IPT	2	173	1.2%
Safety outcomes	SAC	IPT	20	454	4.4%
Safety outcomes	Coiling alone	IS	4	173	2.3%
Safety outcomes	SAC	IS	32	454	7.0%
Safety outcomes	Coiling alone	Rebleeding	6	173	3.5%
Safety outcomes	SAC	Rebleeding	9	454	2.0%
Safety outcomes	Coiling alone	Rerupture	2	173	1.2%
Safety outcomes	SAC	Rerupture	4	454	0.9%

RROC follow-up percentages use patients with available angiographic follow-up as the denominator. Safety outcomes use the full treatment-group denominator.

Supplementary Table S10. Exploratory ATO-Weighted Subgroup Analyses

Outcome	Subgroup	Level	N	OR (95% CI)	P	P for interaction
Discharge mRS 3-6	Bifurcation	No	206	1.043 (0.396-2.749)	0.928	0.558
Discharge mRS 3-6	Bifurcation	Yes	404	0.745 (0.386-1.437)	0.364	
Follow-up mRS 3-6	Bifurcation	No	206	0.880 (0.277-2.793)	0.819	0.655
Follow-up mRS 3-6	Bifurcation	Yes	404	0.674 (0.248-1.829)	0.422	
Initial RROC II vs I	Bifurcation	No	188	0.593 (0.239-1.471)	0.242	0.985
Initial RROC II vs I	Bifurcation	Yes	384	0.597 (0.328-1.086)	0.088	
Initial RROC III vs I	Bifurcation	No	139	0.942 (0.259-3.429)	0.924	0.228
Initial RROC III vs I	Bifurcation	Yes	285	0.357 (0.168-0.758)	0.01	
Follow-up RROC II vs I	Bifurcation	No	156	0.211 (0.052-0.849)	0.03	0.291
Follow-up RROC II vs I	Bifurcation	Yes	306	0.504 (0.236-1.075)	0.074	
Follow-up RROC III vs I	Bifurcation	No	139	0.515 (0.151-1.754)	0.27	0.533
Follow-up RROC III vs I	Bifurcation	Yes	272	0.333 (0.163-0.680)	0.004	
Rerupture	Bifurcation	No	206	0.440 (0.033-5.968)	0.518	0.589
Rerupture	Bifurcation	Yes	404	0.984 (0.221-4.375)	0.982	
Rebleeding	Bifurcation	No	206	0.195 (0.035-1.078)	0.06	0.135
Rebleeding	Bifurcation	Yes	404	0.669 (0.185-2.422)	0.525	
IPT	Bifurcation	No	206	Not calculable		
IPT	Bifurcation	Yes	404	2.018 (0.750-5.432)	0.156	
Ischemic stroke	Bifurcation	No	206	6.518 (0.830-51.154)	0.072	0.484
Ischemic stroke	Bifurcation	Yes	404	2.214 (0.494-9.912)	0.284	
CVS	Bifurcation	No	206	1.780 (0.259-12.224)	0.538	0.751
CVS	Bifurcation	Yes	404	2.169 (0.615-7.647)	0.216	
All-cause mortality	Bifurcation	No	206	1.193 (0.215-6.619)	0.832	0.866
All-cause mortality	Bifurcation	Yes	404	0.992 (0.270-3.641)	0.99	
Discharge mRS 3-6	Size10	<10 mm	571	0.770 (0.482-1.228)	0.258	
Discharge mRS 3-6	Size10	>=10 mm	39	Not calculable		
Follow-up mRS 3-6	Size10	<10 mm	571	0.655 (0.278-1.543)	0.317	
Follow-up mRS 3-6	Size10	>=10 mm	39	Not calculable		
Initial RROC II vs I	Size10	<10 mm	544	0.599 (0.331-1.084)	0.087	
Initial RROC II vs I	Size10	>=10 mm	28	Not calculable		
Initial RROC III vs I	Size10	<10 mm	403	0.706 (0.376-1.328)	0.266	
Initial RROC III vs I	Size10	>=10 mm	21	Not calculable		
Follow-up RROC II vs I	Size10	<10 mm	446	0.340 (0.195-0.594)	<0.001	0.446
Follow-up RROC II vs I	Size10	>=10 mm	16	0.942 (0.026-34.000)	0.968	
Follow-up RROC III vs I	Size10	<10 mm	393	0.414 (0.202-0.849)	0.018	0.742
Follow-up RROC III vs I	Size10	>=10 mm	18	0.621 (0.027-14.409)	0.713	
Rerupture	Size10	<10 mm	571	0.579 (0.124-2.694)	0.47	
Rerupture	Size10	>=10 mm	39	Not calculable		
Rebleeding	Size10	<10 mm	571	0.510 (0.104-2.511)	0.391	0.504
Rebleeding	Size10	>=10 mm	39	0.144 (0.004-5.028)	0.244	

Outcome	Subgroup	Level	N	OR (95% CI)	P	P for interaction
IPT	Size10	<10 mm	571	5.543 (1.761-17.451)	0.005	
IPT	Size10	>=10 mm	39	Not calculable		
Ischemic stroke	Size10	<10 mm	571	3.140 (1.297-7.601)	0.013	
Ischemic stroke	Size10	>=10 mm	39	Not calculable		
CVS	Size10	<10 mm	571	1.897 (0.462-7.800)	0.358	
CVS	Size10	>=10 mm	39	Not calculable		
All-cause mortality	Size10	<10 mm	571	0.968 (0.340-2.757)	0.95	
All-cause mortality	Size10	>=10 mm	39	Not calculable		
Discharge mRS 3-6	WideNeck	No	93	0.723 (0.146-3.579)	0.67	0.874
Discharge mRS 3-6	WideNeck	Yes	517	0.826 (0.455-1.498)	0.512	
Follow-up mRS 3-6	WideNeck	No	93	2.752 (0.228-33.244)	0.398	0.176
Follow-up mRS 3-6	WideNeck	Yes	517	0.603 (0.257-1.414)	0.232	
Initial RROC II vs I	WideNeck	No	86	1.198 (0.758-1.894)	0.403	0.04
Initial RROC II vs I	WideNeck	Yes	486	0.510 (0.238-1.093)	0.081	
Initial RROC III vs I	WideNeck	No	57	1.793 (0.411-7.819)	0.41	0.086
Initial RROC III vs I	WideNeck	Yes	367	0.406 (0.194-0.847)	0.018	
Follow-up RROC II vs I	WideNeck	No	70	0.477 (0.145-1.575)	0.198	0.586
Follow-up RROC II vs I	WideNeck	Yes	392	0.326 (0.163-0.655)	0.003	
Follow-up RROC III vs I	WideNeck	No	61	1.220 (0.486-3.060)	0.647	0.006
Follow-up RROC III vs I	WideNeck	Yes	350	0.304 (0.159-0.582)	<0.001	
Rerupture	WideNeck	No	93	0.766 (0.037-15.974)	0.853	0.97
Rerupture	WideNeck	Yes	517	0.706 (0.058-8.606)	0.776	
Rebleeding	WideNeck	No	93	0.394 (0.028-5.462)	0.46	0.934
Rebleeding	WideNeck	Yes	517	0.447 (0.091-2.189)	0.306	
IPT	WideNeck	No	93	Not calculable		
IPT	WideNeck	Yes	517	5.228 (1.596-17.129)	0.008	
Ischemic stroke	WideNeck	No	93	Not calculable		
Ischemic stroke	WideNeck	Yes	517	2.647 (0.713-6.298)	0.329	
CVS	WideNeck	No	93	2.051 (0.079-53.065)	0.643	0.941
CVS	WideNeck	Yes	517	1.904 (0.555-6.534)	0.291	
All-cause mortality	WideNeck	No	93	Not calculable		
All-cause mortality	WideNeck	Yes	517	0.759 (0.248-2.323)	0.615	

Subgroup effects are exploratory. Treatment-effect heterogeneity should be interpreted using the interaction P value rather than significance within individual strata. Estimates were labeled not calculable when sparse or empty cells precluded reliable estimation.
